# Letter to the Editor: Professionalism—The Role of Quality Improvement

**DOI:** 10.5041/RMMJ.10219

**Published:** 2015-07-30

**Authors:** Kieran Walsh

**Affiliations:** BMJ Learning, BMJ Publishing Group, Londong, UK

## TO THE EDITOR

Mueller is to be congratulated for a comprehensive and detailed exposition on medical professionalism.^1^ There is no question but that professionalism is important—however, Mueller is correct to point out the complexities of the subject and the fact that there is no single or simple way to teach or assess professionalism.

One topic of importance within the professionalism domain is quality improvement. Mueller mentions this subject but could perhaps have made more of it. At its core, professionalism should be about physicians using valid and reliable means to measure the quality of care that they deliver and using evidence-based means of improving the quality of such care.^2^,^3^

In traditional medical curricula, quality improvement used to receive only passing mention. However, in modern curricula quality improvement is integrated with other clinical and non-clinical subjects that professionals must learn. No professional’s practice can be perfect—so anything that can help all professionals improve their practice should also be able to help them with their professionalism. The advantage of stressing the importance of quality improvement is that it is tangible and practical. By contrast, at times the concept of professionalism can appear esoteric or academic. Quality improvement gives the professional something to actually work on—something that should improve them as professionals and at the same time improve outcomes for their patients.

Another advantage of viewing professionalism through the prism of quality improvement is that it encourages the professional to consider team activities. Quality improvement is a team activity and so engenders the concept of the professional as a member of the team—as opposed to the isolated professional leader who makes decisions alone. In the past too many courses concentrated on the leader as an individual with an individual learning style and an individual leadership style. However, recent thinking is that learning styles are evanescent concepts and individual leadership styles less than helpful—largely because most important things happen in teams.^4^–^6^

Quality improvement can also be linked to the assessment of professionals. A good professional is one who continually evaluates the quality of care that they deliver and who continually takes measures to improve quality improvement. Assessments and appraisals of physicians should take this into account as a core component of good practice. Assessments, however, must be sufficiently sophisticated to be able to distinguish the role of the individual in a quality improvement activity and that of the team. An ideal professional is one who takes a leadership role that is appropriate to their experience and that enables all team members to improve their practice for the benefit of patients.

The concept of professionalism must be disseminated to all health care professionals—regardless of grade or specialty—and quality improvement has the potential to play a key role in such dissemination.
